# Development of Highly Sensitive Raman Spectroscopy for Subnano and Single-Atom Detection

**DOI:** 10.3390/molecules26165099

**Published:** 2021-08-23

**Authors:** Yuansen Tang, Naoki Haruta, Akiyoshi Kuzume, Kimihisa Yamamoto

**Affiliations:** 1 Laboratory of Chemistry and Life Science, Tokyo Institute of Technology, Yokohama 226-8503, Japan; tangyuansen@hotmail.com; 2 JST-ERATO, Yamamoto Atom Hybrid Project, Tokyo Institute of Technology, Yokohama 226-8503, Japan; haruta.naoki.3r@kyoto-u.ac.jp; 3 Fukui Institute for Fundamental Chemistry, Kyoto University, Kyoto 606-8103, Japan; 4 Clean Energy Research Centre, University of Yamanashi, Yamanashi 400-8510, Japan

**Keywords:** Raman spectroscopy, surface plasmon resonance, single-atom catalyst, nanostar

## Abstract

Direct detection and characterisation of small materials are fundamental challenges in analytical chemistry. A particle composed of dozens of metallic atoms, a so-called subnano-particle (SNP), and a single-atom catalyst (SAC) are ultimate analysis targets in terms of size, and the topic is now attracting increasing attention as innovative frontier materials in catalysis science. However, characterisation techniques for the SNP and SAC adsorbed on substrates requires sophisticated and large-scale analytical facilities. Here we demonstrate the development of an ultrasensitive, laboratory-scale, vibrational spectroscopic technique to characterise SNPs and SACs. The fine design of nano-spatial local enhancement fields generated by the introduction of anisotropic stellate-shaped signal amplifiers expands the accessibility of small targets on substrates into evanescent electromagnetic fields, achieving not only the detection of isolated small targets but also revealing the effects of intermolecular/interatomic interactions within the subnano configuration under actual experimental conditions. Such a development of “in situ subnano spectroscopy” will facilitate a comprehensive understanding of subnano and SAC science.

## 1. Introduction

Nanomaterials are one of the most widely used functional substances underpinning modern innovation in science and technology, and have numerous applications, such as in nonlinear optics, electronics, catalysts, drug delivery, and biosensors. Distinctive properties and performances of these nanomaterials relative to bulk materials arise from the downsizing of the materials to the nanoscale, thereby inducing a high surface ratio, confined quantum effects, and distortion in crystal structures. Recently, ultrafine particles, so-called subnano-particles (SNPs), are receiving attention as one of the next-generation substances, whose physical, chemical, and electronic properties change discretely by the number of constituent atoms rather than by the size of the particles [1,2,3]. Furthermore, a single-atom catalyst (SAC) is the ultimate substance in terms of size, and arguably the most innovative frontier in the field of inorganic chemistry and catalyst science [4,5]. However, the greatest barrier in the research and development of these subnano and single-atom substances is the difficulty of direct spectroscopic detection and characterisation of these materials due to their poor signal intensity, far below the detection level of current analytical techniques. In particular, the atomic resolution microscopic and structural measurements for characterisation of an SAC requires large-scale facilities equipped with sophisticated instrumentations, including aberration-corrected scanning transmission electron microscopy (AC-STEM), X-ray absorption fine structure spectroscopy (XAFS), and tip-enhanced Raman spectroscopy (TERS). However, spectroscopic studies of SAC are almost unfeasible due to a lack of sensitivity of the conventional approaches.

**Figure 1:**
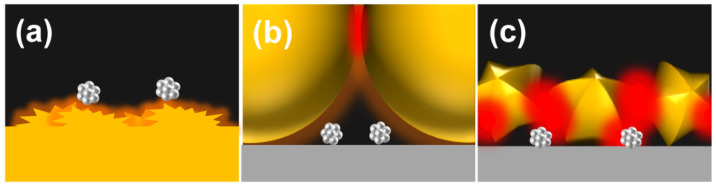
Enhanced Raman spectroscopy for the detection of subnano-particles. Schematics of (**a**) surface-enhanced Raman spectroscopy at a roughened metallic surface, (**b**) optimised shell-isolated nanoparticle enhanced Raman spectroscopy using 120 nm diameter Au nanoparticles as optical amplifiers, (**c**) enhanced Raman spectroscopy with shell-isolated nanostars which produce an abundant proportion of hotspots at the surface that allow detection of single molecules and small particles.

Surface-enhanced Raman spectroscopy (SERS) provides vibrational and chemical “fingerprint” information to identify target molecules with high accuracy, and thus, is a powerful laboratory-scale analytical tool used in various fields, such as material characterisation, biological and chemical sensing, and cancer detection [6,7,8,9,10,11,12]. The enhancement of the SERS effect is explained by two enhancement mechanisms, namely, electromagnetic enhancement and chemical enhancement [13,14]. The former effect arises from the excitation of the localised surface plasmon at roughened or nano-structured metal surfaces (typically Au and Ag) by the incident light (Figure 1a) [13]. This process results in the formation of an evanescent electromagnetic field, which enhances the surface Raman signal intensities substantially. The enhancement by the localised electromagnetic field decays exponentially with distance from the surface. Therefore, SERS is a surface-sensitive analytical technique. However, SERS is not applicable for surface analyses in some conditions: for instance, the case where target substances alter their intrinsic properties by adsorption on the nano-structured surfaces that induces decomposition or by alloying with surfaces, or where the metal surfaces are not coinage metals without nano-structured morphology, whereby negligible enhancement effect are generated.

The invention of the shell-isolated nanoparticle enhanced Raman spectroscopy (SHINERS), one of the most innovative SERS developments [15], overcame the crucial problems of conventional SERS techniques. In this method, Au nanoparticles (NPs) with a diameter of ca. 55 nm were coated with a thin and pinhole-free chemically inert shell and were used as an optical amplifier. The thin and pinhole-free inert shells not only inhibit chemical and electrical contacts between the amplifiers and the target molecule but also prevent self-aggregation of the NPs. This configuration allows the use of any substrate material and morphology of choice since the enhancement takes place at the gap between adjacent shell-isolated nanoparticles (SHINs) (Figure 1b). In this respect, the introduction of the SHINERS technique allows precise characterisation of materials and high versatility measurement on any substrate material [16,17]. However, the measurements of subnano-sized materials are below the detection limit of conventional SERS and SHINERS methods. We have recently reported an extremely sensitive SHINERS method using shell-isolated Au@Ag core-shell nanoparticles as optical amplifiers, demonstrating the first ever detailed vibrational characterisation of metal oxide SNPs achieved by improving the enhancing properties of SHINs, thus boosting the sensitivity of SHINERS [18].

Here, we focus on the development of a laboratory-scale direct spectroscopic method that can provide fingerprint vibrational information of SNPs and single-atom substances adsorbed directly on the substrate with high sensitivity. The key concept to the present work is in the challenge to enhance the surface sensitivity of SHINERS achieving subnano and single-molecule resolution, and this relies heavily on the fine nano-spatial design of the evanescent electromagnetic fields (hotspots) formed at the gap between two SHINs. In practice, the enhancement properties of SHINERS need to be quantitatively evaluated and optimised based on the consideration of the size and shape of the SHINs, together with the thickness of the silica shell [19]. However, it is important to note that a simple increase in the enhancement capability of individual amplifiers is not sufficient to achieve our main goal, i.e., acquiring spectral information from the target SNPs and SACs adsorbed directly on the substrate.

Size-dependent enhancement properties of optical amplifiers have been studied theoretically by the three-dimensional finite-difference time-domain (3D-FDTD) simulation at the gap between two spherical Au NPs, and the highest enhancement was found for the bare Au NPs with a diameter of 120 nm [20]. However, in this case, the hotspots emerged at the gap more than 50 nm above the surface (Figure 1b). Therefore, target molecules, the size of a few nanometres which are adsorbed on the substrate such as SNPs and a SAC, cannot reach the hotspots to “bask” in the strong electromagnetic fields. In other words, it is not only a matter of the potential enhancing capability of individual Au amplifiers, but also the accessibility of the target samples in the hotspots, which is critically important nanotechnology for achieving our goal. Furthermore, what is important here is to directly observe small samples adsorbed on the substrate as it is. Mounting the samples onto the surface of the amplifier is not suitable as an in situ observation method to elucidate the original spectral features of samples, because this configuration would change the experimental environment of samples that affect crucially the electrical and catalytic properties of the samples, such as the substrate effect.

The next key consideration is how to ensure that target molecules on substrates settle into the right position on the substrate within the hotspots. A precise design for the distribution and localisation of the hotspots on the substrate is a key criterion for high sensitivity measurements with high reproducibility. First, the practical location of the hotspots should be close to the substrate surface so that the target molecules are easily accessible to the distributed hotspots near the amplifiers. Second, the distribution of hotspots under laser irradiation should be widespread over the substrate; this will result in a high number of enhanced Raman scattering events, thus realising high detection sensitivity. It has been demonstrated that the presence of sharp edges and tips provides large enhancement of the electromagnetic field at the surface of the amplifiers [21,22,23]. Given these considerations, we conceived that a rational design for the enhancing fields can be realised by fabricating smaller SHINs with multiple apices on their surfaces, generating multiple strong hotspots close to the substrate surface (Figure 1c).

In this study, we established a sensitive laboratory-scale analytical method based on the principles of SERS. The key factor in determining the high sensitivity of SERS is in the successful design of shell-isolated anisotropic stellate-shaped Au NPs, which generate an abundant proportion of nano-spatial hotspots all over the surface. The optimised Raman spectroscopy then allows us to acquire detailed vibrational information of isolated single molecules adsorbed on SNPs and SACs, successfully providing a proof of concept. In addition, this technique was further applied in the in situ configuration under practical reaction conditions. Here, we specifically focused on the Pt surfaces. In addition to ornaments, Pt has been widely studied as an (electro)chemical catalyst due to its unique chemical and electrical properties. In particular, Pt-group metal catalysts are indispensable for the electrode catalyst of the polymer membrane fuel cells.

## 2. Results and Discussion

### 2.1. Fabrication and Characterisation of Anisotropic Au SHINs

A variety of anisotropic Au NPs, including rods, cubes, prisms and dendritic nanoparticles, have already been reported to be fabricated by using different types of surfactants and capping agents during the growth process of the NPs [24,25]. In this work, a classical seed-mediated synthetic method for the synthesis of stellate-shaped NPs (nanostars), developed by Liz-Marzán et al. and featuring PVP as a capping agent in DMF solution, was applied [26,27]. A variety of Au nanostars were synthesised in a systematic manner to study the influence of the structure of the nanostars on the Raman enhancement properties.

**Figure 2:**
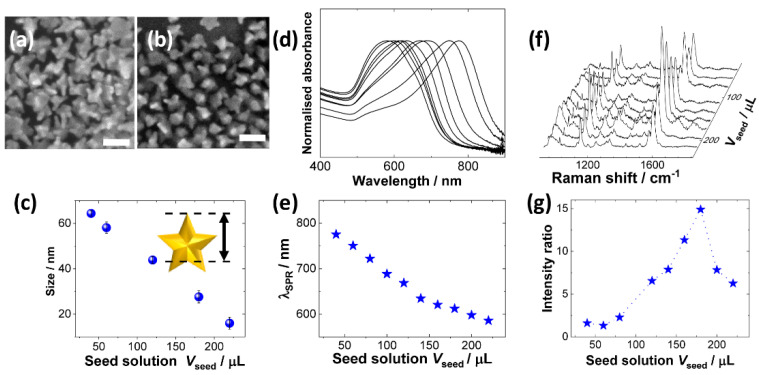
Characterisation of PVP-protected Au nanostars. SEM images of PVP-protected Au nanostars synthesised by different amounts of the seed solution (*V*_seed_). (**a**) *V*_seed_ = 40 and (**b**) 140 μL, respectively (scale bar: 50 nm). (**c**) The particle size, acquired based on the measurement of the longest length from the tip apex to the opposite core surface, was plotted as a function of *V*_seed_, where the error bar shows the standard deviation of the size distribution. (**d**) A series of UV-Vis spectra of Au nanostars synthesised with different *V*_seed_, and (**e**) SPR peak positions (λ_SPR_) as a function of *V*_seed_. (**f**) A series of Raman spectra for a dendrimer sample with shell-isolated Au nanostars with different *V*_seed_ and (**g**) the corresponding signal intensity at 1580 cm^−1^ as a function of *V*_seed_, normalised with that from the blank spectrum acquired in the absence of the Au nanostar.

SEM images showed that the as-prepared Au nanostars have multiple needle structures, and their average size decreased with increasing amount of the injected seed Au NP solution (*V*_seed_) into the growth solution (Figure 2a–c). It is important to note that a high concentration of PVP was found to give reproducible synthesis of the nanostars with exceptionally high yield, as demonstrated by the SEM images, where no other shapes other than nanostars were observed.

A series of UV-vis spectra for the as-prepared Au nanostars using different *V*_seed_ were recorded to evaluate the effect of *V*_seed_ on the optical properties (Figure 2d). Given that the Au nanostars have an anisotropic structure, the absorption spectra exhibited two broad peaks in the visible and near-infrared regions: a shoulder peak centred around 530 nm was due to the surface plasmon resonance (SPR) from the central part of the Au core. The additional major peak in the near-infrared region was attributed to the longitudinal SPR peaks from the elongated tip structures of the nanostars. The latter peak was blue-shifted from 780 to 580 nm with increasing *V*_seed_ (Figure 2d). This result indicated that the SPR optical properties of the Au nanostars were tuneable by controlling the mixing ratio of the seed and the growth solutions (Figure 2e), which is extremely important for the optimisation of SHINERS sensitivity because the coupling of the SPR peak position and the excitation laser wavelength defines the optical enhancement properties in the SERS measurement.

The Raman enhancement for the series of Au nanostars prepared with different *V*_seed_ was evaluated after coating with a silica shell. The shell coating process is almost the same as that for spherical SHINs [15], where the heating time was fixed at 20 min to form pinhole-free and thin silica layers of 2–3 nm thickness on average (see Supporting Information S1). The highest Raman enhancement was recorded for shell-isolated Au nanostars prepared with *V*_seed_ = 180 µL (Figure 2f,g), of which the SPR peak appeared at 605 nm in the corresponding UV-vis spectrum (Figure 2e). Subsequent TEM observations revealed the average size of the optimised Au nanostars was 28 ± 2 nm (Figure 2c). It is fair to note that the synthesis method of shell-isolated nanostars shown here is not a new method, but the experimental conditions were optimised according to the goal of the present research, that is, to demonstrate the ultimate optimisation in sensitivity aimed at developing a spectroscopic technique that enables direct observation of subnano and single-atom substances adsorbed directly on the substrates.

### 2.2. Enhanced Raman Sensitivity with Shell-Isolated Au Nanostars

**Figure 3:**
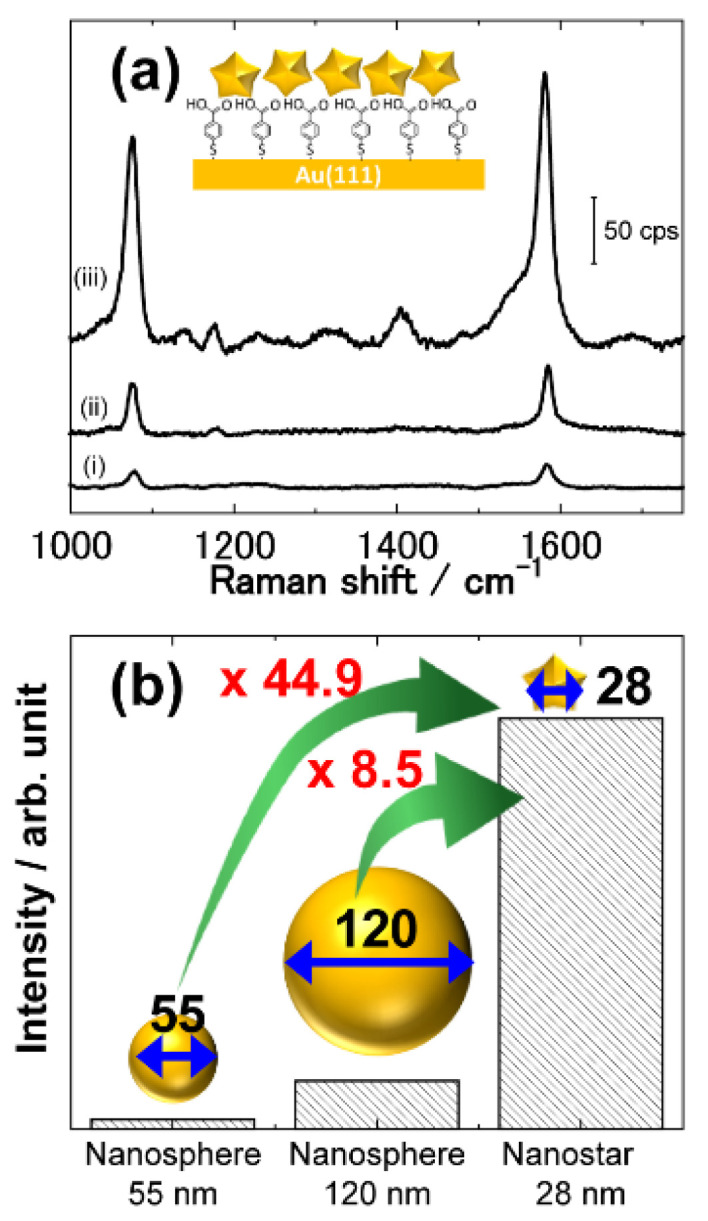
Enhancement effect of shell-isolated Au nano-structured amplifiers. (**a**) Raman spectra of mercaptobenzoic acid self-assembled monolayer (MBA-SAM) on Au(111) enhanced by (i) 55 nm Au nano-spheres, (ii) 120 nm Au nano-spheres and (iii) 28 nm Au nanostars. The excitation wavelength was 632.8 nm. (**b**) Comparison of Raman signal intensities of MBA-SAM on Au(111) with different optical amplifiers calculated for the peak intensity at 1583 cm^−1^ which was assigned to the ring breathing mode.

The enhancement properties of the newly developed shell-isolated Au nanostar amplifiers were evaluated using a self-assembled monolayer of mercaptobenzoic acid (MBA-SAM) formed on an Au(111) surface as a standard sample. The corresponding Raman spectra revealed anomalous enhancement factor of nanostar of 1.4 × 10^9^, having practical Raman intensity signals some 44.9 and 8.5 times higher than that of the nano-sphere SHINs with diameters of 55 and 120 nm, respectively (Figure 3).

For optimisation of the optical amplifiers in SERS techniques, it is essential to tune the SPR peak position of the amplifiers with respect to the excitation wavelength, as described before. In the case of nanostars, two SPR peaks appeared which originated from the central part of the nanostars and from the elongated tip structures of the nanostars, respectively. The latter peak appears in the near-infrared range and the degree of the shift depends on the size of the apices. This peak has been used in application studies with laser excitation in the near-infrared range which causes less damage to target samples during irradiation. Therefore, nanostars with long apices have been investigated extensively in the biosensors and bio-detection technology fields. However, in this study, it is of primary importance to fabricate small optical amplifiers for the design of the hotspots generated close to the substrate surface for the sake of subnano-material detection (as shown in Figure 1c). Therefore, the size of the optical amplifier should be as small as possible, and thus, we persisted in using the excitation laser of wavelength 632.8 nm instead of that in the near-infrared range for coupling with the SPR absorption of small apices on the nanostars amplifiers.

### 2.3. SHINERS Study of Pyridine Molecules Adsorbed on Pt Subnano-Islands

**Figure 4:**
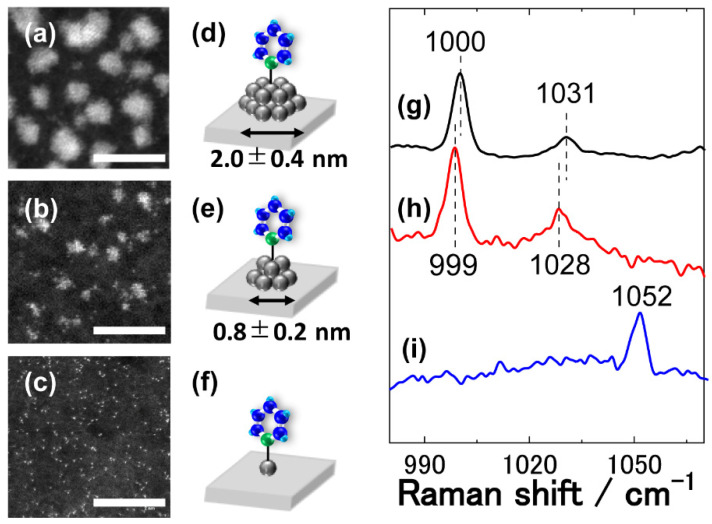
Raman studies for the Py molecules on the Pt subnano-islands and Pt single-atoms with shell-isolated Au nanostars. (**a**–**c**) TEM images of Pt subnano-islands prepared by APD (scale bar: 5 nm), (**d**–**f**) schematic images and (**g**–**i**) SHINER spectra of Py molecules adsorbed on the Pt islands and atoms. Pt subnano-islands were prepared by (**a**,**d**,**g**) 100, (**b**,**e**,**h**) 10 and (**c**,**f**,**i**) 1 APD pulses, respectively. The former two samples had the average sizes of (**a**) 2.0 ± 0.4 and (**b**) 0.8 ± 0.2 nm, respectively, while the latter sample gave individually deposited atoms on the HOPG surface.

Historically, pyridine (Py) has been used in Raman measurements as a probe molecule to obtain surface information as well as a standard sample in a new Raman technique for comparison purposes with the conventional SERS techniques [12,13,14]. In this study, Pt islands of size range of subnano-metre scale down to a single atom were prepared by the arc plasma deposition (APD) technique on highly ordered pyrolytic graphite (HOPG) substrates under vacuum, where Py was subsequently adsorbed as the probe molecule (Figure 4). The size of the Pt subnano-islands was adjusted in the range from 0.8 to 2.0 nm in diameter by controlling the number of APD pulses (Figure 4a–c; more details given in Supporting Information S2).

Py molecules were adsorbed strongly on the Pt atoms via the N-end in the upright configuration with σ-type bonding via the lone-pair electrons of nitrogen and the unoccupied orbital of the Pt (Figure 4d–f). Interaction between Py and the Pt surface causes charge transfer from Py to the Pt surface, leading to a change in the polarisability and the electronic properties of the Py molecules that significantly affect the Raman spectral features. In this respect, it was our intention to use Py as a probe to detect the binding interactions between Py and the Pt subnano-islands as a function of island size, thus monitoring a subtle size-dependent transition in the electronic properties of the subnano-scale Pt islands.

The Raman spectra revealed two peaks from the Py adlayers that were assigned to the ring breathing modes (ν_1_) at 999–1000 cm^−1^ and the deformation modes (ν_12_) at 1028–1031 cm^−1^, respectively (Figure 4g,h) [28], while no signal was detected for the conventional SHINERS method using 55 nm Au spherical SHINs (data not shown). It is a new finding that the peaks in question shifted to the lower wavenumber region (red shift) with decreasing the size of the Pt islands (see detail in Supporting Information S3). Given that the peak shift of the ν_1_ mode was rather less sensitive to the size of the Pt subnano-islands, hereafter, we only discuss the spectral features of the ν_12_ mode as an indicator of the strength of the Py-Pt bond, and thus of the electronic properties of the subnano-Pt islands.

Wu et al. performed a detail SERS study of Py adlayers on roughened bulk metal surfaces (Ag, Au and Pt) [29,30]. The Raman signals of the ν_12_ mode were observed at 1036 and 1038 cm^−1^ on Ag and Au surfaces, respectively, while this signal on Pt was diminished. The authors rationalised this slight blue shift of the ν_12_ mode on Au and Ag with respect to pure Py liquid (1029 cm^−1^) by the formation of Py-M bonds (M = Ag, Au) [29,30]. Density functional theory (DFT) calculation studies showed that the distance between a nitrogen atom of Py and a metal surface atom followed the order Pt < Au < Ag [28]. The authors indicated that the strong binding interaction between Py and Pt lead to a diminution in the Raman intensity of the ν_12_ mode [30]. This suggests that this vibration mode on the Pt surface should appear, if detectable, at a higher wavenumber than 1038 cm^−1^ due to the stronger interaction of Pt with Py than that on Au, which is apparently inconsistent with our observation where broad peak assigned to the ν_12_ mode on the Pt subnano-islands appeared at 1028–1031 cm^−1^ (Figure 4g,h). In the literature, a red shift of the adsorbates in the spectral band was ascribed by several reasons, such as stronger d-π* back donation between adsorbates and metal surface [31], and increase in the surface coverage of adsorbates [32]. However, the red shift of the ν_12_ mode in the case of subnano-islands may be derived from some other reasons.

A first direct spectroscopic observation of subnano materials was reported on tin oxide (SnO_x_) subnano-particles [18], revealing a broad Raman signal in the spectra with a significant red shift from that of bulk SnO_2_, which could not be ascribed to any of the modes predicted by the group theory. On the other hand, Sn oxide nanoparticles with sizes of 2 to 5 nm exhibited sharp signals from the bulk SnO_2_. A red shift and broadening of the peak were apparent only with subnano-particles. The emergence of a broad signal is typically indicative of the accumulation of signals from Sn-O bonds with slightly different binding configurations. Together with the loss of a sharp signals from the bulk SnO_2_, complete deformation of the crystalline structure of rutile SnO_2_ was suggestive of the formation of multiatom clusters with distorted atomic configuration in the subnano-particles [18]. Therefore, we believe the significant red shift of the ν_12_ mode of Py on Pt subnano-islands was derived from the specific electronic states, exclusively expressed in the subnano structures, induced by the distortion of the crystal structure.

### 2.4. Detection of the “Fingerprint” of a Single Atom

Atomically dispersed single Pt atoms were deposited on the HOPG substrate by tuning the experimental conditions in the APD (Figure 4f). Two vibrational peaks were observed at 1012 and 1052 cm^−1^ for Py molecules adsorbed on single atoms of Pt on the HOPG surface (Figure 4i), although the former peak was slightly unclear due to low S/N ratio. A DFT calculation study showed that the ν_1_ and ν_12_ modes for Py_1_-Pt_1_ appeared at 1009.4 and 1053.1 cm^−1^, respectively [28], in good agreement, surprisingly, with our experimental results. The substantial blue shift of the ν_12_ mode of nearly 24 cm^−1^ compared to that for the Py-on-Pt subnano-islands (0.8 nm) (Figure 4h) was explained by the absence of Pt-Pt inter-atomic interactions in the case of a single Pt atom [28], where the absence would significantly strengthen the binding interaction between Py and Pt, inducing considerable blue shift in spectra. Although it was not clear why the peak intensity of the ν_1_ mode was noticeably smaller than that of the ν_12_ mode, a good accordance in frequencies is apparent between experimental results and a DFT calculation. Further DFT studies indicated that Py adsorbed on a Pt dimer (Pt_2_) revealed the ν_12_ modes at 1039 cm^−1^ [28], but this signal was not observed no matter how many times it was repeated at different locations on the HOPG in our experiments. The absence of the Py-Pt_2_ signals suggested that the majority of Pt atoms were adsorbed as monatomic atoms, as observed in the corresponding TEM images (Figure 4c).

Observation of isolated molecules adsorbed on the Pd single atoms have just been reported in 2021 by the group that invented the original SHIENRS method [33], where the Pd atoms were adsorbed on the surface of the Au@TiO_2_ amplifiers. On the other hand, with the nanostar amplifiers, we achieved in this study a direct observation of isolated molecules adsorbed on Pt single atoms located on the substrate, simulating real environment of SAC. Establishing an ultrahigh sensitive, laboratory scale spectroscopic technique capable of direct detection of spectral properties that reflect specific electronic states of single atoms in the real reaction environment is a major update of “in situ spectroscopy”, facilitating a comprehensive understanding of subnano and SAC science.

**Figure 5:**
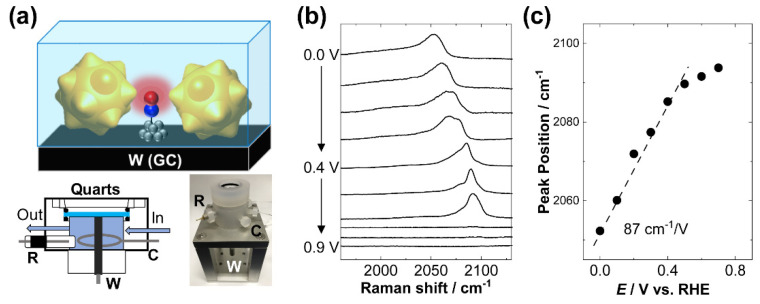
In situ Raman study of CO adsorbed on Pt islands with shell-isolated Au nanostars: (**a**) schematics of in situ shell-isolated nanostar enhanced Raman spectroscopy and the homemade cell. (**b**) Electrode potential-dependent steady-state SHINER spectra for CO adsorbed on Pt islands (0.9 nm in diameter) in CO-saturated 0.1 M HClO_4_, and (**c**) potential dependent Raman peak frequency shifts of the CO stretching mode. The Stark tuning rate was 87 cm^−1^/V.

It is important to note that one of the greatest advantages of SHINERS is that one can perform measurements in electrochemical/chemical/gaseous environments, thus, affording in situ/operando real-time monitoring. In particular, the SHINERS method combined with an electrochemical setup can provide real-time information on catalytic reactions (Figure 5a), hence not only identifying the reaction intermediates and reaction mechanisms, but also detecting the structural and chemical changes that catalysts themselves undergo during the catalytic process [16,17,34,35,36].

Figure 5b shows the potential-dependent CO stretching Raman spectra for the adsorbed species on Pt subnano-islands with an average diameter of 0.9 nm, revealing a blue shift with a Stark slope of 87 cm^−1^/V (Figure 5c). This value is notably higher than that for a bulk Pt electrode (32 cm^−1^/V) [37] and commercial Pt catalysts (38 cm^−1^/V), reflecting a transition of the electronic properties of Pt subnano-islands. This transition of the Stark slope is currently being analysed using DTF calculations with different island sizes to find the main cause of the significant transition of the Stark slope.

## 3. Materials and Methods

### 3.1. Materials

Polyvinylpyrrolidone (PVP) with a molecular weight of 40,000 g/mol were purchased from Sigma-Aldrich (St. Louis, MO, USA), as well as gold(III) chloride trihydrate, sodium citrate, (3-aminopropyl)triethoxysilane (APTES) and sodium silicate. Pyridine, sodium borohydride, hydrochloric acid, *N*,*N*-dimethylformamide (DMF) and methanol were purchased from Kanto Chemical Co. Most of the chemicals are in high grade (>99.5%), while sodium silicate solution and sodium borohydride were at the grade of 27% and 98%, respectively. High quality 70% HClO_4_ (Suprapur grade) for electrochemical measurement was purchased from Merck. All chemicals were used without further purification. Aqueous solutions were prepared with ultrapure water produced by the MilliQ system (Merck Millipore, Burlington, MA, USA, 18.2 MΩcm, TOC < 4 ppb).

### 3.2. Characterisation Methodologies

Shimadzu UV-VIS-NIR Spectrophotometer UV-3600PC (Shimadzu, Tokyo, Japan) was used to acquire UV-vis absorption spectra of Au nanostar solution at 20 °C. UV cells used were disposable plastic cells with an optical path length of 10 mm. The wavelength range recorded was 230–900 nm where there is no disturbing absorption from the plastic cell. SEM images were acquired by Hitachi High-Technologies Field Emission Scanning Electron Microscopes S-5500 (Hitachi, Tokyo, Japan). Accelerating voltage was typically at 5 kV. Cu-150P STEM grid with PVB-C15 supporting film (film thickness 30–40 nm) was purchased from Oken-Shoji and used without further cleaning process. High-resolution STEM and TEM images were obtained by the aberration-corrected transmission electron microscope (JEM-ARM200F, JEOL, Tokyo, Japan), with the accelerating voltage at 80 kV. Grids were the same as that used for SEM measurements. Raman spectra were acquired by the Confocal Microscope Laser Raman Spectrometer (Horiba Jobin Yvon LabRAM HR Evolution, Kyoto, Japan). The excitation laser source was 632.8 nm He-Ne laser with the typical power of max. 120 μW on the surface. The spectrometer equipped an 800 mm focal length monochromator with a long working distance objective lens (50× magnification, OLYMPUS). Si(100) substrate for Raman measurements was cleaned by immersing overnight in the H_2_SO_4_ + H_2_O_2_ (4:1) *piranha* solution (very strong oxidising agent and requires special attention when used), followed by excessive rinsing with MilliQ water and drying in Ar stream before each experiment. In addition, freshly cleaved HOPG was also used for Raman measurement substrates. Arc plasma deposition (Advanced Riko) was used to fabricate Pt nanoparticles and subnano-islands on HOPG surfaces as well as on TEM grid. In the APD chamber, an electric arc is used to vaporise Pt material from a Pt cathode target. The vaporised Pt then condenses on either HOPG/TEM grid substrates to form Pt subnano-islands. The capacitance value was fixed at 360 μF during deposition, while the bias voltage and the number of pulses were varied in the range of 70–120 V and 1–100 times, respectively, to find the optimal condition. All the processes were conducted under high-vacuum conditions (1.8 × 10^−3^ Pa), while other preparation processes were carried out under Ar atmosphere in the glove box.

The in situ spectroscopic measurements in electrochemical condition were conducted with a home-made Daiflon cell equipped with Pt wire and an Ag/AgCl electrode as counter and reference electrodes, respectively, as well as two inlets for solution and gas exchange. All potentials in this paper are referred to the RHE scale. All in situ Raman experiments were carried out in CO-saturated 0.1 M HClO_4_ solution in the strict absence of oxygen. A commercial potentiostat (Model-440 CH Instruments, Austin, TX, USA) ensured the simultaneous electrochemical control.

### 3.3. Synthesis of Au Nanostars

The synthetic process for the Au nanostars was based on a seed-mediated method, whereby Au NPs (diameter 5–7 nm) were first synthesised as a seed, then covered by an anisotropic Au overlayer. Polyvinylpyrrolidone (PVP) was used as a surfactant for the nanostars, crucially important for the growth of the anisotropic stellate-shaped Au particles. In short, Au seeds were prepared by mixing HAuCl_4_ solution (90 mL, 0.01 wt%), sodium citrate solution (2 mL, 1 wt%) and freshly prepared NaBH_4_ solution (1 mL, 0.075 wt%), and then stirring the mixed solution at room temperature overnight. Then, 50 mL of the as-prepared seed solution was mixed with 5 g of PVP at room temperature for 24 h to acquire the PVP-coated Au seed solution (see Supporting Information S4).

Second, 160 μL of 1 wt% HAuCl_4_ solution was mixed with 3 g PVP in 15 mL DMF, where differing amounts of PVP-coated seed solution (*V*_seed_: 40–500 μL) were later added and with stirring at 35 °C for 12 h to allow anisotropic growth of tips on the surface of the seed NPs forming a stellate shape. The subsequently formed nanostar solution was mixed with a 10 mL of methanol and then centrifuged at 8000 rpm for 1 h, where the supernatant was later replaced by a mixture of ultrapure water and methanol (1:1) to facilitate removal of the organic solvent and excess surfactant. The centrifugation process was repeated five times to clean the Au nanostar samples.

### 3.4. Synthesis of Shell-Isolated Au Nanostars

In a 50 mL round-bottom flask, 30 mL of each PVP-coated Au nanostar solution was first added to 0.4 mL of 10 mM 3-Aminopropyltriethoxysilane (APTES) aqueous solution and the mixture was stirred vigorously at room temperature for 15 min. Then, 2 mL of 0.54 wt% sodium silicate solution (pH adjusted to 10.2–10.5 by addition of dilute HCl solution) was added and the solution was stirred for 3 min. Thereafter, the flask was placed in a boiling water bath and the solution was vigorously stirred. The reaction was terminated after specified reaction times (10–60 min) by cooling the solutions in an ice bath [38]. Finally, the sample solution was centrifuged at 7500 rpm for 15 min and the supernatant was replaced by ultrapure water. The centrifugation process was repeated at least three times to obtain clean shell-isolated Au nanostars.

## 4. Conclusions

In the quest to undertake detailed characterisation of subnano and SAC materials and demonstrate new applications, optimisation of the Raman spectroscopic technique was achieved by the effective and rational design of the fine nano-spatial plasmon-induced local electromagnetic fields. Introduction of anisotropic optical amplifiers and implementation of the systematic characterisation processes for the amplifiers in terms of size and shape facilitated a fine-tuning of the local SPR features, not only generating pronounced enhancement capability of amplifiers in the Raman scattering, but also improving the accessibility of the target molecules at the hotspots, achieving sensitivities 45 times higher than that of conventional methods. The high sensitivity of the Raman techniques with shell-isolated Au nanostars now enables a direct observation of weak Raman signals from isolated probe molecules adsorbed on the subnano-islands and single atoms with high sensitivity, accuracy and reproducibility with surface selectivity, revealing new chemical information associated with intermolecular, interatomic and molecule–atom interactions. Furthermore, it should be emphasised that the results for the direct detection and characterisation of extremely small materials were realised with laboratory-scale equipment with the capability for in situ measurements.

The fundamental understanding of reaction processes enables direct interpretation of structure-activity correlation to be realised in a way that is not accessible by standard ex situ methods, thus providing a template for further development of novel subnano and single-atom (electro)catalysts for electrolysis, fuel cells and battery applications. Furthermore, it is anticipated that such advanced spectroscopy techniques with single-atom sensitivity will become powerful characterisation tools when combined with a state-of-the-art scanning transmission electron microscopy, X-ray absorption spectroscopy and advanced modelling and simulation in computation chemistry, facilitating an in-depth understanding of newly developed, innovative materials.

## Supplementary Materials

The following are available online. Figure S1: Silica coating of nanostars, Figure S2: Arc plasma deposition to fabricate subnano-scale Pt islands on HOPG, Figure S3: SHINERS studies of adsorbed Py on the subnano-scale Pt islands, Figure S4: Characterisation of the PVP-coated seed Au nanoparticles.

## Sample Availability

Samples of the compounds are available from the authors.
