# Supplementary material for: Development of Highly Sensitive Raman Spectroscopy for Subnano and Single-Atom Detection
Source: Molecules. 2021 Aug 23;26(16):5099. doi: 10.3390/molecules26165099 (PMC8400086; doi:10.3390/molecules26165099)
Supplement: Supplementary file 1 [file molecules-26-05099-s001.zip › molecules-1348843-supplementary.pdf]

Supporting Information

Development of a highly sensitive Raman spectroscopy for the subnano and single-atom detection

Yuansen Tang[1], Naoki Haruta[2,3], Akiyoshi Kuzume[2,4*], Kimihisa Yamamoto[12*]

[1] Laboratory for Chemistry and Life Science, Tokyo Institute of Technology, Yokohama 226-8503, Japan

[2] JST-ERATO, Yamamoto Atom Hybrid Project, Tokyo Institute of Technology, Yokohama 226-8503, Japan

[3] Fukui Institute for Fundamental Chemistry, Kyoto University, Kyoto, 606-8103, Japan

[4] Clean Energy Research Centre, University of Yamanashi, Kohu, 400-8510, Japan

*Correspondence to : yamamoto.k.at@m.titech.ac.jp, akuzume@yamanashi.ac.jp

Table of Content

S1: Silica coating of nanostars

S2: Arc plasma deposition to fabricate subnano-scale Pt islands on HOPG

S3: SHINERS studies of adsorbed Py on the subnano-scale Pt islands

S4. Characterisation of the PVP-coated seed Au nanoparticles

S1: Silica coating of nanostars

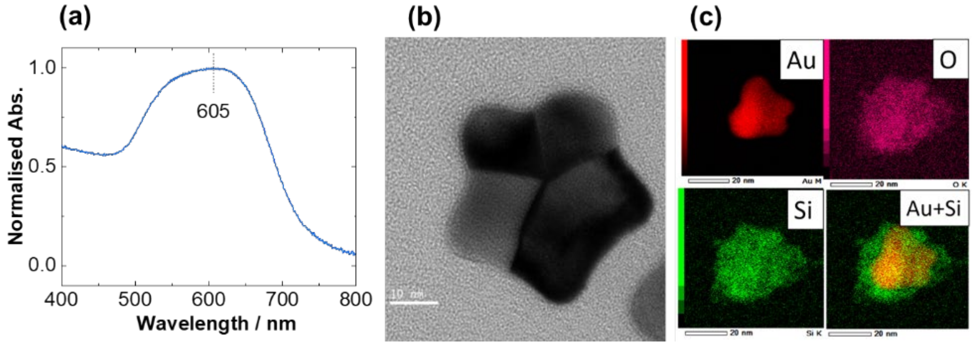

Figure S1: Characterisation of shell-isolated Au nanostars. (a) A selected UV-vis spectrum that showed SPR peaks at 610 nm for shell-isolated Au nanostars. (b) TEM and (c) EDS mapping images of shell-isolated Au nanostars prepared with 40,000 g/mol PVP, followed by 30 min of reaction time to coat with 2-3 nm thick silica shell without pinhole.

Pin-hole test was carried out to make sure there was no pin-hole in the silica shell. Shell-isolated Au nanostars were casted on the Si surface and let dried, where 1 mM Py solution was over-casted later. The resulting surface was rinsed with MilliQ water to remove excess physisorbed Py molecules. Then SHINERS measurement was carried out to examine the presence/absence of the pinhole in the silica shell layer. If there is a pinhole in the silica shell, Py molecules immediately spotted the pinhole and adsorbed on the exposed Au surfaces on the nanostars through the pinhole, giving a strong Raman signal of a Py molecule at 1012 cm^{-1} [29], whereas no Raman signal from Py molecule was observed in the absence of pinhole because there was almost no place Py molecules could adsorb (Figure S10).

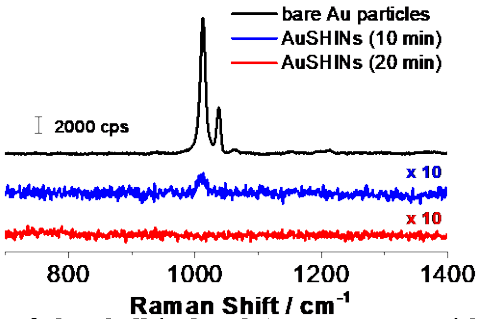

Figure S2: Pinhole test of the shell-isolated Au nanostars with Py probe. SERS and SHINERS studies of Py on bare Au particles (black) and shell-coated Au nanostars with a reaction time of 10 min (blue) and 20 min (red), respectively.

S2: Arc plasma deposition to fabricate subnano-scale Pt islands on HOPG

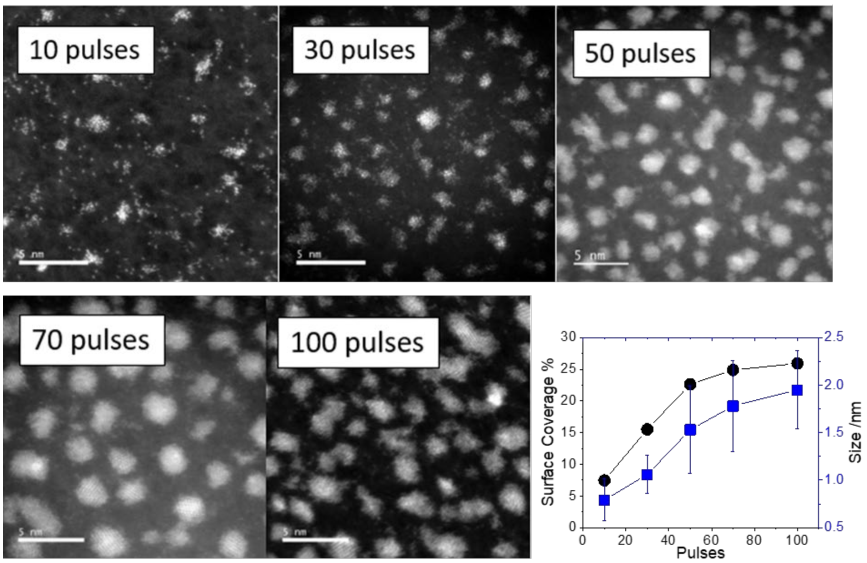

Figure S3: TEM observation of the Pt islands fabricated by the APD. TEM images of Pt islands fabricated by the Arc plasma deposition (APD) directly on the carbon thin films deposited on TEM grids with different numbers of APD pulse, as indicated in each TEM images. The resulting average size together with the surface coverage was plotted in the function of the number of the APD pulse.

S3: SHINERS studies of adsorbed Py on the subnano-scale Pt islands

5 µL of shell-isolated Au nanostar solution was casted on the Pt-islands-modified HOPG substrate, letting the SHINS droplet to dry naturally. Later, 10 µL of Py solution (10 mM) was cast-and-dried, followed by a rinsing with Milli-Q water to remove excess physisorbed Py. 632.8 nm He-Ne laser was used for the excitation light resource (Figure S12a). Peak positions and full-widths at half maximum were plotted in the function of Pt island size, showing a gradual decrease of both values with decreasing Pt size, indicative of the isolation of Py molecule from neighbours (Figures S12b and S12c).

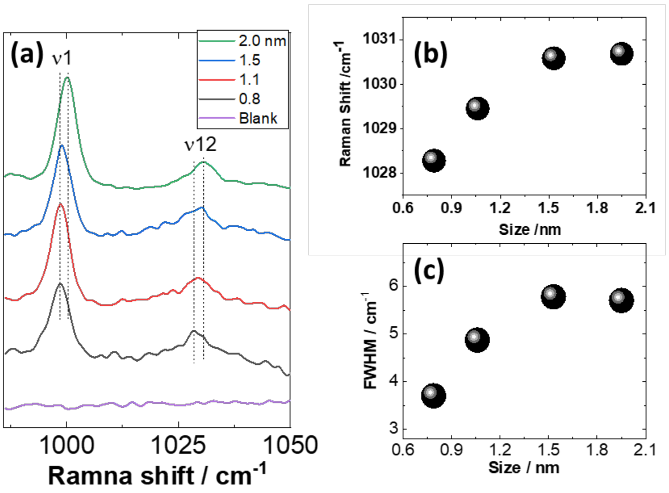

Figure S4: SHINERS study of Py adsorbed on subnano-scale Pt islands. (a) SHINER spectra of Py adsorbed on subnano-scale Pt islands with different size from 0.8 to 2.0 nm in diameter, and the size dependent plots of (b) peak position of the symmetric deformation mode (ν_{12}) and (c) its FWHM values.

S4. Characterisation of the PVP-coated seed Au nanoparticles

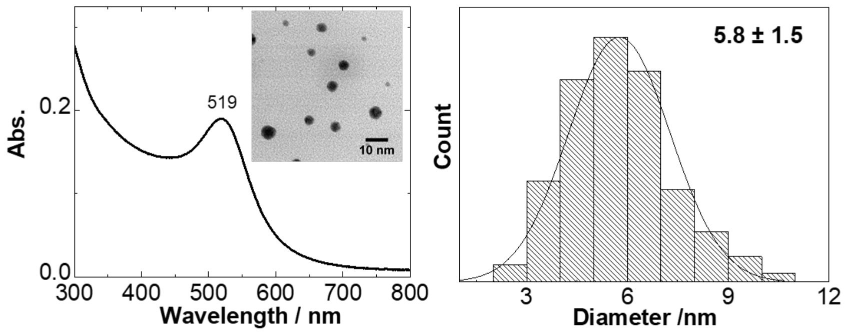

Figure S5: Characterisation of PVP-coated Au seed nanoparticles. (a) A UV-Vis absorption spectrum and a TEM image of as-prepared Au seed nanoparticles and (b) the size distribution histogram obtained by measuring 200 particles in several TEM images, revealing the average diameter of 5.8 ± 1.5 nm.
